# Effect of acustimulation on nausea and vomiting and on hyperemesis in pregnancy: a systematic review of Western and Chinese literature

**DOI:** 10.1186/s12906-016-0985-4

**Published:** 2016-01-13

**Authors:** Els Van den Heuvel, Maria Goossens, Hilde Vanderhaegen, Hai Xia Sun, Frank Buntinx

**Affiliations:** 1 Department of Family Medicine and Primary Health Care, Ghent University, Ghent, Belgium; 2 Department of General Practice, KU Leuven, Leuven, Belgium; 3 Department of Family Medicine and Primary Health Care, Interuniversity Training Centre for General Practitioners, Leuven, Belgium; 4 Department of General Practice, University of Maastricht, Maastricht, Netherlands

**Keywords:** Nausea, Vomiting, Hyperemesis, Pregnancy, Acupressure, Acupuncture, Acustimulation, Moxibustion, Systematic review

## Abstract

**Background:**

Nausea and vomiting in pregnancy (NVP) and hyperemesis gravidarum (HG) have a significant impact on quality of life. Medication to relieve symptoms of NVP and HG are available but pregnant women and their caregivers have been concerned about the teratogenic effect, side effects and poor efficacy. The aim of this review was to investigate if there is any clinical evidence for the efficacy of acustimulation in the treatment of NVP or HG.

**Methods:**

A systematic review of randomized controlled trials (RCTs), including both English and Chinese databases was conducted to assess the efficacy of various techniques of acustimulation for NVP and HG. The methodological quality of the studies was assessed using the Cochrane’s risks of bias tool. Revised STRICTA (2010) criteria were used to appraise acustimulation procedures. Pooled relative risks (RRp) and standard mean deviations (SMD) with 95 % confidence intervals (CI) were calculated from the data provided by the investigators of the original trials.

**Results:**

Twenty-nine trials including 3519 patients met the inclusion criteria. Twenty trials could be included in statistical pooling. The overall effect of different acustimulation techniques shows a significant reduction for the combined outcome for NVP or HG in pregnancy as a dichotomous variable (RRp 1.73, 95 % CI 1.43 to 2.08). Studies with continuous outcome measures for nausea, vomiting and the combined outcome did not show any evidence for relieving symptoms of NVP and HG (SMD −0.12, 95 % CI −0.35 to 0.12).

**Conclusions:**

Although there is some evidence for an effect of acustimulation on nausea and vomiting or hyperemesis in pregnancy, results are not conclusive. Future clinical trials with a rigorous design and large sample sizes should be conducted to evaluate the efficacy and safety of these interventions for NVP and HG.

## Background

Nausea and vomiting in pregnancy (NVP) is commonly experienced in early pregnancy, most frequently between 6 and 12 weeks. NVP can continue till 20 weeks, and persist after this time for up to 20 % of women [1]. Prevalence of nausea ranges from 50 to 80 %. Prevalence of vomiting and retching is around 50 %. Persistent and severe nausea and vomiting may lead to malnutrition and the development of hyperemesis gravidarum (HG), a disorder that may cause the loss of >5 % of original body weight, dehydration, electrolyte imbalance, acidosis or ketosis during pregnancy [2]. HG is less common, affecting between 0.3 and 3 % of pregnant women [1]. In China, HG prevalence rates range from 0.35 to 0.47 % [3].

NVP has a significant impact on quality of life for pregnant women and their families [4]. It causes discomfort, disability and suffering and results in absence from work and social activities [5]. Furthermore, almost 50 % of women reported that NVP negatively affected the relationship with their partner and their partner’s daily life [4]. Therefore, it is important to treat this condition [6].

Both pharmaceutical and non-pharmaceutical [1] remedies for NVP have been suggested. Pharmaceutical treatments include anticholinergics, antihistamines, dopamine antagonists, vitamins (B6 and B12), H3 antagonists [1], corticosteroids and metoclopramide. After the thalidomide tragedy in the 1960s, pregnant women and their caregivers have been concerned about the use of pharmaceutical interventions to control or relieve symptoms during pregnancy. Besides the possible teratogenic effect and side effects such as drowsiness, sedation, heartburn or arrhythmia [1, 7], poor efficacy of pharmaceutical medications [8] used in the past has left a therapeutic gap in the treatment of nausea and vomiting during pregnancy [9]. Women are commonly offered psychological support [5], dietary advice and advice about the (usually) self-limiting nature of the condition [1].

In recent years, the use of complementary and alternative therapies has become popular in many Western countries [10]. These include herbal remedies (ginger, chamomile, peppermint, raspberry leaf), homeopathic remedies (Nux vomica, Pulsatilla), acupressure, acustimulation bands, acupuncture [1, 6, 7] and moxibustion [11–13]. Pregnant women may perceive these as “natural” and therefore safe.

In China, acupuncture has been used to treat morning sickness for thousands of years [5].

A number of studies of various acupuncture modalities have assessed their efficacy for treating NVP and HG [14]. The latest Cochrane review [1] considered studies of acupressure randomized against sham acupuncture or other controls. The overall conclusion was that evidence regarding effectiveness of acustimulation of the PC6 point and of auricular acupressure was limited. Acupuncture showed no significant benefit for women in pregnancy. Festin [15] reported that acupressure may be more effective than sham acupressure in reducing NVP. However, evidence was weak, and interventions and outcomes varied between trials. It thus remained unclear whether acupuncture is more effective than sham acupuncture in reducing NVP and whether acupressure and acupuncture are effective in treating HG.

Although a number of systematic reviews on the effect of acustimulation for NVP have recently been performed [1, 15–18], theyonly included a single article published in Chinese. Moreover, moxibustion was only included in one previous review [1]. Given the fact that many studies of acupuncture and moxibustion for NVP and HG have been published in non-Western scientific literature and have not been reviewed, the literature identified by previous reviews may not be comprehensive enough to cover all current evidence. Therefore, we performed a comprehensive systematic review on randomized controlled trials of acustimulation for NVP and HG published in both Chinese and Western language literature. The aim of this review was to investigate if acupressure, acupuncture or moxibustion, together called acustimulation, were more effective than sham or placebo acupuncture or other conventional treatments in the treatment of NVP and HG [19].

## Methods

### Search strategies

A comprehensive electronic search was performed in the following databases from their inception to August 2014: Cochrane Database of Systematic Reviews, The Cochrane Central Register of Controlled Trials (CENTRAL), Medline (National Library of Medicine), Embase and Science Direct (Elsevier), Latin American and Caribbean Health Sciences (LILACS), Allied and Complementary Medicine Database (AMED), Database of abstracts of reviews of effects (DARE), Trip Database, Web of science core collection database, Cumulative Index to Nursing and Allied Health Literature (Cinahl), Physiotherapy Evidence Database (Pedro), BJI Best Practices in OvidSP, BMJ Clinical evidence and National Institute for Health and Care Excellence (NICE). An additional search for articles in the Chinese language was performed in the Chinese Biomedical Literature Database (CBM), China National Knowledge Infrastructure (CNKI), VIP database (Chinese Scientific Journals database), WanFang database, Index to Chinese Periodicals of Hong Kong (HKInChiP), Chinese Clinical Trial Register (ChiCTR), and ProQuest Digital Dissertations (PQDD). The latter focuses on so-called “gray literature”, such as unpublished studies, dissertations and conference reports.

The following terms were used in the search strategies: (acupuncture or acupressure or needle or auricular acupuncture or acupoint stimulation or moxibustion) and (pregnan*) and (nausea or vomiting or morning sickness or hyperemesis). Mesh terms were used as much as possible. Equivalent Chinese terms were used in searching the Chinese language databases.

### Inclusion criteria

#### Study selection

One author, fluent in both English and Chinese, searched the databases and assessed potentially relevant articles against the inclusion criteria. Any doubt regarding the eligibility of a study was discussed within the team.

#### Types of studies and subjects

Inclusion of studies was restricted to randomized controlled clinical trials (RCT) or quasi-randomized clinical trials (qRCT) with at least 20 participants per arm, studying women suffering from NPVor HG in normal pregnancy and for whom acupressure, acupuncture, auricular stimulation or moxibustion was used as treatment. We used no restriction for the women’s age or for gestational age. Nausea and vomiting as a result of pregnancy complications such as partum hemorrhage, hypertension, pre-eclampsia, diabetes in pregnancy or cesarean section were excluded from this review. We did not include observational studies (cohort, case control, case study), studies reported in abstracts only and studies with a cross-over design without a wash-out period of at least one week because of the time effect reported in some previous studies [20, 21].

#### Language

Studies in Arabian or Farsi were excluded from this analysis.

#### Types of interventions

According to the principles of traditional Chinese medicine (TCM), relief of nausea and vomiting is accomplished by stimulation of meridian points to restore the balance of “Qi” flow affecting digestive functions.

#### Acupuncture

Acupuncture is defined as the stimulation of an acupoint with a needle. The definition also extends to auricular acupuncture and electro-acupuncture, both using needle penetration. Other variants of acupuncture, such as acupoint injection, laser acupuncture, acupotomy (small needle-scalpel), and transcutaneous electrical nerve stimulation (TENS) were excluded.

#### Acupressure

Acupressure is a gentle, noninvasive form of stimulation achieved by applying pressure to acupuncture points [4, 22]. Traditional Asian systems use a number of acupuncture points for anti-emetic treatments. The PC6 or NeiGuan point is a major site for relief of nausea and vomiting. In earlier studies this site was termed P6, but following WHO standard acupuncture nomenclature we have changed P6 into PC6 [23]. PC6 is located on the volar side of the wrist approximately 3 cm above the wrist crease, between 2 easily palpated tendons. Pressure can be applied manually (using fingers or thumbs) or with wristband devices that provide steady pressure from a small button or disc on the site. SeaBand is one example of a commercially developed wristband device [14]. Studies using other points for acupressure or auricular acupressure were also included in the review.

#### Moxibustion

Moxibustion is defined as the stimulation of acupoints with heat generated by burning of moxa (*Artemisia Vulgaris* L.). Usually, a moxa cigar is kept about 2–3 cm above the skin.

We also included studies combining acupuncture and moxibustion treatment (acupuncture and moxibustion combined, AMC), which is usually performed by placing a moxa block on the handle of the acupuncture needle.

Studies using acustimulation in combination with other treatments, such as medication, massage, physiotherapy, traditional Chinese herbs, or injection were excluded, as was cupping.

#### Types of control interventions

We included studies that used sham or placebo acupuncture, IV fluid therapy, oral Western medication, Chinese herbal medicine or no treatment as control intervention.

Sham acupressure involves needling or applying pressure in a minimal way such as needling real or wrong points or non-points shallowly with minimal stimulation. Critics of sham needling suggest that even minimal needling produces some physiological effects and is not a truly physiologically inert procedure. Placebo acupuncture uses a non-inserted needle with a telescopic function or a needle encased in a cartridge so that the patient cannot tell whether the needle has been inserted or not. Unlike sham acupuncture, placebo acupuncture is presumed to provide an almost physiologically inert placebo [24, 25].

Although acupoint specificity was not the focus of this review, we also included studies that compared the same intervention with different combination of acupoints.

#### Types of outcome measures

In this review, we limited our analyses to primary outcomes: (cure or improvement of) NVP, or reduction of ketones in case of HG. In most studies, the severity of NVP episodes was measured by commonly used, validated instruments such as the Rhodes Index score or a visual analogue scale (VAS). The Rhodes index consists of three subscales: nausea, vomiting (both with a range of 0 to 12) and retching (range 0 to 8) [1]. The visual analogue scale (VAS) includes a 10 cm ruler with a beginning and an end, and a clear range allowing patients to indicate their health condition. Zero represents the best condition (lack of nausea) and ten represents the worst possible degree of nausea [22].

In other studies, outcome was reported as a reduction or cessation in nausea, vomiting, retching, ketonuria, Outcomes were mainly classified into the following categories: cured, improved or ineffective. “Cured” referred to complete relief of nausea and vomiting and disappearance of ketones in case of HG at the end of the treatment period or during follow-up. “Improved” indicated overall relief of nausea and vomiting and disappearance of ketones for HG, but with occasional reoccurrence of symptoms. “Ineffective” referred to no improvement. Because the “cured” category appeared to be the only consistent category across these studies in assessing treatment efficiency, this review categorized the cured rate into cured or not cured.

We did not include analyses on secondary outcomes e.g. rate of food intake, length of inpatient stay, weight gain, inpatient parenteral drug and fluid use because of a wide variation in outcome measures between different studies.

#### Adverse outcomes

If they were available, data on side effects of the interventions were extracted.

### Data extraction

One author (EVdH) extracted the data and two other assessors (HXS and HV) checked the extracted data. Discrepancies were resolved through discussion or, if required, a second review author was consulted. If information regarding any of the above was unclear, we contacted authors of the original reports to provide further details.

For each study, the following variables were extracted: study design, number of arms, population, gestational age, outcome measures, interventions and intervention details.

### Quality assessment

#### Reporting of interventions in controlled trials of acupuncture

We used the “Revised Standards for Reporting Interventions in Clinical Trials of Acupuncture (STRICTA): Extending the CONSORT Statement” criteria. The items of the STRICTA checklist are acupuncture rationale, details of needling, treatment regime, other components of treatment, practitioner background and control intervention [26].

#### Bias risk assessment

The methodological quality of the identified studies was independently assessed by three authors. One author (EVdH) assessed bias risk for each study while two others (HXS and HV) assessed the Chinese and English studies, using the Cochrane’s risks of bias tool criteria outlined in the Cochrane Handbook for Systematic Reviews of Interventions (Higgins 2011). After cross-checking for accuracy, we resolved any disagreement by discussion or by involving a fourth assessor.

Cochrane’s risks of bias assessment includes the following domains: random sequence generation, allocation concealment, blinding of participants and personnel, blinding of outcome assessment, incomplete outcome data, selective outcome reporting and other sources of bias. Each domain was rated as “low”, “high”, or “unclear”.

Given the impossibility of blinding the acupuncturist, we only assessed the blinding of participants and personnel on the type of intervention a participant received. We assessed the methods as low risk of bias for single blinding, due to the nature of the intervention. Blinding was assessed as high risk in the following cases: no blinding, more than two active intervention arms and blinding of treatment type without blinding of the control condition (no intervention). Incomplete outcome data (attrition bias) were assessed as low risk if no outcome data were missing or if outcome data were missing in less than 20 % in each arm of the study. Reasons for missing data were reported and balanced across groups.

### Statistical analysis

The main analyses focused on the results from dichotomous outcomes presented as a relative risk (RR), and continuous outcomes presented as the mean outcome on the last intervention day, both with a 95 % confidence interval (CI). Besides these, we performed subgroup analyses per acustimulation technique and per outcome measure (cure and improvement of nausea and vomiting). To test for heterogeneity, the bull-eye test (carefully studying the forest plots) and the I^2^ test were performed for both main analyses and subgroup analyses when calculating summary statistics. An I^2^ test > 50 % was considered to indicate a moderate or high level of heterogeneity. In pooling studies with continuous outcomes we only included those that reported at least a mean and SD or SE from each group. A random-effect analysis was performed in view of the high level of heterogeneity between studies. If a study had more arms, we used the control group that provided the most optimal degree of blinding. All statistical analyses were performed using STATA version13 (reference: StataCorp. Stata Statistical Software: Release 13, College Station, TX: StataCorp LP. 2013.)

## Results

### Study selection

**Fig. 1:**
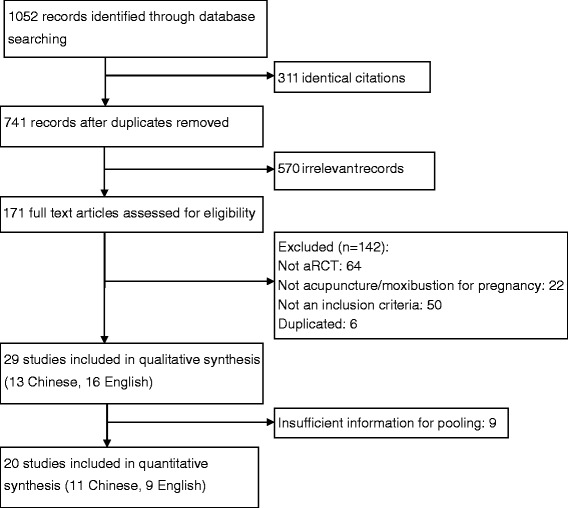
Flow chart of study selection

The search identified 1052 potentially relevant citations for review. After removal of duplicates, 741 citations were left. Of these, 507 papers were excluded for reasons of irrelevance and 171 full-text articles retrieved for further assessment. Of these, 29 studies met the inclusion criteria and were included in this review. Nine studies were excluded from pooling because of insufficient information. Finally, 20 studies were included in quantitative analyses (Fig. 1).

### Description of included studies

Of the 29 studies, 16 were published in English and 13 were conducted in China and published in Chinese. All studies were full-length journal reports. All recruited pregnant woman with symptoms of nausea with or without vomiting. There were 15 studies on nausea and vomiting alone (13 English, 2 Chinese). HG was considered in 14 studies (3 English, 11 Chinese). Nine studies [2, 3, 12, 27–32] were performed in a hospital, one study [33] involved in- and outpatients and in two studies [13, 34] it was not clearly reported whether patients were hospitalized or not. In the remaining 17 studies all subjects were outpatients. Together, these studies involved 3519 subjects with 1431 in the treatment arm and 2088 in the control arm. Eighteen trials used a two-armed parallel group design, 10 studies a three-armed and one study [10] a four-armed parallel group design.

The sample size of the studies included ranged from 55 [35] to 593 [10] subjects. Gestational age at the time of recruitment varied. Most studies reported on women in the first trimester of pregnancy (less than 12 weeks gestation). Three studies recruited women up to 30 weeks [2, 9, 20] and in one study [11] women with a gestational age of more than two months were included, but the upper limit was not specified [1].

The included studies examined acupressure, acupuncture, auricular acupressure and moxibustion. Study duration varied from four to ten days in 25 studies. Four studies lasted two to four weeks [5, 8, 10, 35]. Treatment frequency varied from once a day to once every week. The duration of each treatment session lasted between five and twenty minutes for acupuncture or moxibustion and up to 8–24 h continuously for acupressure. Table 1 presents the characteristics of all included studies.

**Table 1:** Baseline characteristics of the studies

1st author, year	Design, number of arms	Population (n), gestational age	Outcomes	Participants (n), intervention		dose, frequency, treatment duration
Country				Treatment group	Placebo control group	
Acupressure finger						
Dundee 1988 Ireland [36]	qRCT, 3	350, 6–14 w	NVP	119, PC6	112, sham, dummy point near right elbow	5 min every 4 h, 4 days
					119, control: no treatment	
Belluomini 1994 California, US [37]	RCT, 2	60, ≤ 12 w	NVP	30, PC6	30, sham, placebo point	10 min, 4×/d, for 7 days
Shin 2007 South Korea [2]	qRCT, 3	66, 5–30 w	HG	23, PC6 + IV fluid therapy	21, sham control, a bony part around the radial pulse + IV fluid therapy	10 min, 3×/d before meal from day 2 - day of discharge (mean 5–7 days)
					22, control, only conventional IV fluid therapy	
Rad 2012 Iran [22]	RCT, 2	80, first trimester	NVP	40, pressure thumb of researcher on the two symmetrical KID21 points + Vit B 6 40 mg	40, pressure thumb of researcher on a false point + Vit B 6 40 mg	20 min/d for 4 days + Vit B 6 40 mg
Jiang 2012 China [3]	RCT, 2	130, 5–10, 7 w	HG	65, PC6 + IV fluid therapy + ear acupressure stomach, spleen, duodenum, liver, shen men, heart	65, IV fluid therapy	10 min, 3×/d before meal of nausea for 7 days
						Control: 3–7 days
Acupressure band						
O’Brien 1996 Canada [9]	RCT, 3	161, 4.6–23.6 w	NVP	54, PC6	53, sham: acupressure band inappropriately placed 54, control: no treatment	Band applied for 5 days, removed morning of day 6
Norheim 2001^a^ Norway [38]	RCT, 2	97, 8–12 w	NVP	48, PC6	49, placebo, wristband with felt patch, no button PC6	24 h/d, 4 day run-in, 4 day intervention, 4 day follow-up, 12d
Steele 2001 Michigan, US [39]	qRCT, 2	110, ≤ 13 w	NVP	68, PC6	42, placebo, PC6 without acupressure buttons	Continuously on both wrists for 4 days, remove only when bathing
Werntoft 2001 Sweden [5]	RCT, 3	60, mean 10 w	NVP	20, PC6	20, sham: button on upper side of wrist	On for 24 h, only not when showering, for 14 days
					20, control: no treatment	
Heazell 2006 Australia [27]	RCT, 2	80, 5–14 w	HG	40, PC6	40, sham: a site on the dorsal aspect of the forearm	8 h a day, from 9 AM to 5 PM, length of inpatient stay (mean 3.4)
Jamigorn 2007 Thailand [40]	RCT, 2	66, 6–12 w	NVP	33, PC6 + placebo tablets	33, sham: wristband on dummy-point + 50 mg tablets of Vit B6	Bands: continuously for 5 days
						Tablets: every 12 h for 5 days
Can Gurkan 2008 Turkey [20]	QRCT, 3	75, 5–20 w	NVP	26, PC6	24, sham: upper side wrist	Daytime, taken off at night; no bands day 1–3, bands on day 4–6, no bands day 7–9, for 9 days
					25, control: no treatment	
Saberi 2013 Iran [6]	RCT, 3	143, ≤ 16 w	NVP	48, PC6	50, ginger caps	Nothing on day 1–3, treatment on day 4–7
					45, control: no treatment	PC6: continuously bilateral
						Ginger caps: 3×/day
Acupuncture						
Knight 2001 UK [35]	RCT, 2	55, 6–10 w	NVP	28, needling PC6, St 36, Ren 12, SP4, St 44	27, cocktail sticks on bony regions near acupoint	Needles left during 15 min, 2× in first week, then 1×/ week for 2 weeks
Smith 2002 Australia [10]	RCT, 4	593, ≤ 14 w	NVP	148, maximum 6 various points based on TCM diagnosis	148, PC6	Needles left during 20 min, 2× in first week, then weekly, for 4 weeks.
					148, sham: close to acupoints	
					149, control: no treatment	
Neri 2005 Italy [8]	RCT, 2	81, ≤ 12 w	HG	43, needling PC6, CV12, ST 36, + acupressure PC6	38, metoclopramide infusion + Vit B12 complex(30 mg/day)	Acupuncture: 20 min, 2×/ week + acupressure for 6–8 h/day, for 2 weeks.
						Metoclopramide: infusion 2×/week + vit. B12, for 2 weeks.
Zhang 2005 China [26]	RCT, 3	150, 6–12 w	HG	50, needling + moxibustion CV12, PC6, ST36, SP9	50, Chinese drug group:	Acup: 10–15 min, 2×/d, for 7 days
					Suye Huanglian decoction	Chinese drug: 2×/d, 7d
					50, Western medicine: IV fluid therapy + phenobarbital	Western drug: daily, 7d
Liu 2007 China [29]	RCT, 2	94, early pregnancy	HG	47, needling: scalp, stomach area, CV12, PC6, ST36 + IV fluid therapy	47, control: IV fluid therapy	1×/d for 10 days
Wang 2008 China [34]	RCT, 2	95, early pregnancy	HG	53, CV17, CV12, SP6, PC6, ST36	42, control: IV fluid therapy	Acupuncture: 30 min, 1×/d, 6d
						Control: 1×/d, 6d
Mao 2009 China [33]	RCT, 3	90, 6–12 w	HG	30, IV fluid therapy + needling BL11, ST37, PC6, SP4, CV12, ST36	30, Western medicine: IV fluid therapy + luminal 30 mg	Each group IV fluid therapy Acup: 25 min, 2×/d for 7 days
					30, Chinese drug group: IV fluid therapy + Chinese herbal decoction	Western medicine: 3×/d for 7 days
						Chinese drug group: 3×/d for 7 days
Liu 2011 China [41]	RCT, 2	60, early pregnancy	HG	30, needling CV12, PC6, ST36	30, moxibustion ST36, CV12, PC6, SP4	15–20 min, 1×/d for 10 days
Ma 2013 China [30]	RCT, 2	60, early pregnancy	HG	30, CV12, BL21 + IV fluid therapy	30, IV fluid therapy	Acupuncture: 20 min, 1×/d for 5 days
						IV fluid therapy: 1×/d, for 5 days
Auricular acupressure						
Ou 2001 China [42]	RCT, 3	90, early pregnancy	NVP	Group 1: 30, ear acupressure: diaphragm (bilateral), shen men, kidney + Chinese herbal medicine	30, group 2: ear acupressure: diaphragm (bilateral), shen men, kidney	Group 1: acupressure: 10 min, 3×/d + herbs 3×/d, for 7 days
					30, group 3: Chinese herbal medicine	Group 2: acupressure: 10 min, 3×/d, for 7 days
						Group 3: herbs: 3×/d for 7 days
Puangsricharern 2008 Thailand [7]	RCT, 2	91, ≤ 14 w	NVP	45, magnet pellets, placed at both auricles	46, no treatment, 6d	30 s, 4×/day before meals and at bedtime, day 3-day 6
Li 2010 China [31]	RCT, 3	141, 5–30 w	HG	47, ear acupoints: stomach, spleen, duodenum, liver, shen men, heart,+ needling CV 12, PC6, ST36	47, PC6 acupressure	Ear acupressure: 3×/d before meals or in case of nausea
					47, IV fluid therapy	Acupuncture: 30 min, 1×/d for 10 days
						PC6 acupressure: 10 min, 3×/d before meals or nausea, for 10 days
Liu 2012 China [32]	RCT, 2	54, mean 8 w	HG	27, pylorus, stomach, spleen, esophagus, duodenum, liver, heart, subcortex, shen men, jiao gan.	27, fasting for 2–3 d, rest,	Bilateral, 2 min, 15 min before meal, 3×/d for 7 days
					IV fluid therapy	
Moxa						
Fan 1995 China [11]	RCT, 2	302, >2 m	NVP	151, moxa SP6, CV4 ST36, Li3	151, chinese herbal decoction	5–10 min, 1×/d for 7 days
						Herbs:1×/d for 7 days
Xu 2009 China [12]	RCT, 2	51, early pregnancy	HG	26, IV fluid therapy + moxa ST36, CV12, PC6, SP4	25, IV fluid therapy	Moxa: 15–20 min, 1–2×/d
						IV fluid 1×/d, for 10 days
Lu 2012 China [13]	RCT, 2	64, 38–80 d	HG	32, IV fluid therapy + citicoline 500 mg + moxa ST36, CV12, PC6	32, IV fluid therapy + citicoline 500 mg	IV fluid: 1×/d
						Moxa: 20 min, 2×/d for 5 days

*NVP* Nausea and vomiting during pregnancy, *HG* Hyperemesis gravidarum; ^a^Based on the review van Helmreich [4]: *n* = 48/49

The effectiveness of acupressure was examined in 13 studies. Eight studies [2, 5, 9, 20, 22, 27, 36, 37] compared acupressure against sham acupressure, two studies [38, 39] used a placebo control group, and four studies compared with no treatment [5, 6, 9, 20]. One study [40] compared acupressure to vitamin B6 50 mg. In this study, women in both groups also received a placebo intervention. One study compared the use of acupressure with ingestion of ginger capsules [6]. One study [38] only presented the number per group in percentage in the results tables. Based on another study [4], we used *n* = 48/49.

All of these studies examined the result of an intervention with acupoint PC6 (Nei Guan) using finger or wrist band, except for two [3, 22]. Of these, one trial [3] compared the PC6 point in combination with auricular acupressure, and one trial [22] compared acupressure on the KID21 (You Men) point on the abdomen with sham acupressure on the abdomen. In this study, all women had also taken 40 mg vitamin B6 twice daily. Patients suffering from HG had also received IV fluid therapy in both arms of two studies [2, 3].

Nine trials [8, 10, 28–30, 33–35, 41] examined the effectiveness of acupuncture. They examined the result of an intervention using a variety of different acupoints according to TCM, except for two studies [8, 28]. Of these, one trial [8] added acupressure on the PC6 point between acupuncture sessions in the treatment group and one trial combined the needling with moxibustion [28]. Two trials compared acupuncture with sham acupuncture [10, 35]. In one of these [10], separate groups received traditional, PC6, sham acupuncture or no treatment. Acupuncture was compared to conventional or herbal interventions in three trials [8, 28, 33], to IV fluid therapy in three studies [29, 30, 34] and to moxibustion in one study [41].

Four studies [7, 31, 32, 42] compared auricular acupressure to Chinese herbs [42], no treatment [7] and IV fluid therapy [31, 32]. One study with IV fluid therapy [31] combined ear acupressure with acupuncture needling and also had one arm comparing ear acupressure with PC6 acupressure. In the study [7] comparing ear acupressure to no treatment, patients were allowed to take anti-emetic drugs when needed. The authors reported that the results appeared to favor the treatment group, although scores were lower in this group at baseline. Hence, results were difficult to interpret [1].

There were three studies on moxibustion [11–13], comparing moxa to Chinese herbal medicine [11], IV fluid therapy [12] and one study with moxa in the treatment group as the only difference between the two intervention groups.

The studies for acupuncture and moxibustion were mainly conducted in Chinese. They reported on the treatment of HG using a combination of the following five points: Zu San Li (ST36), Nei Guan (PC6), Zhong Wan (CV12), Gong Sun (SP4) and San Yin Jiao (SP6). Studies using the PC6 point alone for acupressure were mainly in English.

### Standards for reporting interventions in clinical trials of acupuncture (STRICTA) in the included studies

Table 2 presents an appraisal of the standards for reporting acupuncture treatment in all the included studies using the revised STRICTA criteria (2010) [26]. None of the included studies reported the acustimulation procedure sufficiently detailed to satisfy STRICTA criteria. Although treatment regimen and control interventions were always reported, details of needling or acupressure and other components of treatment were often insufficiently described. The background of the TCM practitioner was only reported in one study [22].

**Table 2:** Appraisal of acupuncture, acupressure and moxibustion procedure based on the Revised STRICTA (2010)

1st author, year	Acupuncture rationale	Details of needling or acupressure							Treat-ment regimen	Other components of treatment		Practitioner background	Control or comparator interventions
		No. of needle insertions	Points used	Depth of insertion	Responses sought	Needle stimulation	Needle/pressure retention time	Needle or wristband type		Other interventions administered to the acupuncture group	Setting and context		
Acupressure finger													
Dundee 1988	TCM	NA	R	NA	NR	NA	R	NA	R	NR	NR	NR	R
Belluomini 1994	TCM	NA	R	NA	NR	NA	R	NA	R	NR	NR	NR	R
Shin2007	TCM	NA	R	NA	NR	NA	R	NA	R	R	R	NR	R
Rad 2012	TCM	NA	R^a^	NA	R	NA	R	NA	R	R	R	R	R
Jiang 2012	TCM	NA	R^a^	NA	R	NA	R	NA	R	R	R	NR	R
Acupressure band													
O’Brien 1996	TCM	NA	R^a^	NA	NR	NA	R	R	R	NR	NR	NR	R
Norheim 2001	TCM	NA	R^a^	NA	NR	NA	R	R	R	NR	NR	NR	R
Steele 2001	TCM	NA	R^a^	NA	NR	NA	R	R	R	NR	NR	NR	R
Werntoft 2001	TCM	NA	R	NA	NR	NA	R	R	R	NR	NR	NR	R
Heazell 2006	TCM	NA	R^a^	NA	NR	NA	R	R	R	R	R	NR	R
Jamigorn 2007	TCM	NA	R	NA	NR	NA	R	R	R	R	R	NR	R
Can Gurkan 2008	TCM	NA	R^a^	NA	NR	NA	R	R	R	NR	NR	NR	R
Saberi 2013	TCM	NA	R^a^	NA	NR	NA	R	R	R	R	R	NR	R
Acupuncture													
Knight 2001	TCM	R	R^a^	R	R	R	R	R	R	NR	NR	NR	R
Smith 2002	TCM	R	R	R	R	R	R	R	R	NR	NR	NR	R
Neri 2005	TCM	R	R	R	R	R	R	R	R	R	R	NR	R
Zhang 2005	TCM	R	R	NR	R	R	R	R	R	R	R	NR	R
Liu 2007	TCM	R	R	NR	NR	NR	NR	NR	R	R	R	NR	R
Wang 2008	TCM	R	R	R	R	R	R	R	R	NR	NR	NR	R
Mao 2009	TCM	R	R	R	R	R	R	R	R	R	R	NR	R
Liu 2011	TCM	R	R	NR	R	NR	R	R	R	NR	NR	NR	R
Ma 2013	TCM	R	R	R	NR	NR	R	R	R	R	R	NR	R
Auricular acupressure													
Ou 2001	TCM	NA	R	NA	NR	NR	NR	NA	R	NR	NR	NR	R
Puangsricharern 2008	TCM	NA	R^a^	NA	NR	NR	R	R	R	R	NR	NR	R
Li 2010	TCM	R	R	NR	NR	NR	R	R	R	R	R	NR	R
Liu 2012	TCM	NA	R^a^	NA	R	NA	R	R	R	R	NR	NR	R
Moxa													
Fan 1995	TCM	NA	R	NA	R	NR	R	NR	R	NR	NR	NR	R
Xu 2009	TCM	NA	R	NA	R	NR	R	NR	R	R	R	NR	R
Lu 2012	TCM	NA	R	NA	NR	NR	R	NR	R	R	R	NR	R

*TCM* acupoint selection based on Traditional Chinese Medicine Theory, *NA* not applicable, *R* reported, *NR* not reported, R^a^ reported and mentioned if unilateral or bilateral

MacPherson H, Altman DG, Hammerschlag R, Youping L, Taixiang W, White A, Moher D; STRICTA Revision Group. Revised STandards for Reporting Interventions in Clinical Trials of Acupuncture (STRICTA): extending the CONSORT statement. PLoS Med. 2010 Jun 8;7(6):e1000261

### Bias risk assessment in the included studies

Table 3 presents the results of bias assessment risk. The methodological quality of the included studies was mixed. Most of the studies had at least one or two items scoring unclear or high, except for one study [35], which had a low score for all items. With regard to selection bias, three studies [29, 31, 36] were rated at high risk of bias for random sequence generation. More than 80 % of the studies did not describe allocation concealment. The amount of missing outcome data in most of the studies was generally low, with attrition levels below 20 % and the reasons for attrition and missing data well reported. Almost 60 % of studies had a high risk with respect to blinding of participants and personnel, especially in the Chinese studies.

**Table 3:** Cochrane’s risk of bias assessment

1st author (Year)	Random sequence generation	Allocation concealment	Blinding of participants and personnel	Blinding of outcome assessment	Incomplete outcome data	Selective outcome reporting	Other sources of bias
Acupressure finger							
Dundee 1988	High	Unclear	High	Low	High	Low	Unclear
Belluomini 1994	Low	Unclear	Low	Low	High	High	Low
Shin2007	Low	Unclear	Low	Low	Low	Low	Low
Rad 2012	Unclear	Unclear	Low	Low	Low	Unclear	Low
Jiang 2012	Unclear	Unclear	High	Low	Low	Unclear	unclear
Acupressure band							
O’Brien 1996	Low	Low	Low	Low	Low	Unclear	Low
Norheim 2001	Low	Unclear	Low	Low	Low	Unclear	Low
Steele 2001	Low	Low	Low	Low	Low	Unclear	Low
Werntoft 2001	Unclear	Unclear	Unclear	Unclear	Unclear	Low	Unclear
Heazell 2006	Unclear	Unclear	Low	Low	Low	Low	Unclear
Jamigorn 2007	Low	Low	Low	Low	Low	Unclear	Unclear
Can Gurkan 2008	Unclear	Unclear	Low	Low	Low	Unclear	Low
Saberi 2013	Low	Unclear	High	Low	Low	Low	Low
Acupuncture							
Knight 2001	Low	Low	Low	Low	Low	Low	Low
Smith 2002	Low	Low	Low	Low	High	Unclear	Unclear
Neri 2005	Low	Unclear	High	Unclear	Low	Unclear	Unclear
Zhang 2005	Low	Unclear	High	Low	Low	Low	Low
Liu 2007	High	Unclear	High	Low	Low	Low	Unclear
Wang 2008	Low	Unclear	High	Low	Low	Low	Unclear
Mao 2009	Low	Unclear	High	Low	Low	Low	Low
Liu 2011	Low	Unclear	High	Low	Low	High	Unclear
Ma 2013	Unclear	Unclear	High	Unclear	Low	High	Unclear
Auricular acupressure							
Ou 2001	Low	Unclear	High	Low	Low	Low	Low
Puangsricharern 2008	Low	Unclear	High	Low	Low	Low	Unclear
Li 2010	High	Unclear	High	Low	Low	Low	Low
Liu 2012	Unclear	Unclear	High	Unclear	High	High	Unclear
Moxa							
Fan 1995	Unclear	Unclear	High	Unclear	Low	Low	Unclear
Xu 2009	Unclear	Unclear	High	Low	Low	Low	Unclear
Lu 2012	Unclear	Unclear	High	Low	Low	Unclear	Unclear

Higgins JPT, Green S (editors). *Cochrane Handbook for Systematic Reviews of Interventions* Version 5.1.0 (Updated March 2011). The Cochrane Collaboration, 2011. Available from www.cochrane-handbook.org

### Effects of interventions

Outcomes in most Chinese studies [3, 11–13, 28–34, 41, 42] and three English studies [8, 36, 38] were mainly classified into the following categories: cured, improved or ineffective.

Most studies on NVP used only subjective outcome measures such as the Rhodes Index score or Visual Analogue Scale (VAS) questionnaire to assess severity of nausea and vomiting. Objective outcome measurements using ketones for HG were used in five studies [2, 29, 31, 33, 42]. Table 4 shows the data for dichotomous outcomes (cured rate and RR) and Table 5 those of continuous outcomes on the last day of intervention (mean + SD).

**Table 4:** Dichotomous outcomes from original studies (*included in pooling)

Studies (author, year)	Number of subjects, intervention		Outcome measurement	Outcomes cured rate n/N (%)		Included in pooling
	Treatment group	Comparator		Treatment group	Comparator	
Dundee 1988	Acupressure P6 *N* = 119	Sham acupressure *N* = 112	Emetic symptoms: cured rate based on subjective report	32/119 (26.89 %)	17/112 (15.18 %)	*
Dundee 1988	Acupressure P6 *N* = 119	No treatment *N* = 119	Emetic symptoms: cured rate based on subjective report	32/119 (26.89 %)	15/119 (12.60 %)	
Jiang 2012	Acupressure P6 + ear acupressure *N* = 65	Conventional IV fluid therapy *N* = 65	Nausea, vomiting, rate of food intake, ketonuria: Cured rate	42/65 (64.6 %)	25/65 (38.5 %)	*
Norheim 2001	Acupressure P6 *N* = 48	Placebo acupressure *N* = 49	Intensity of symptoms: VAS. Improved rate.	34/48 (71 %)	31/49 (63 %)	*
Neri 2005	Acupuncture + P6 acupressure *N* = 43	Metoclopramide infusion + Vit B 12 complex *N* = 38	Vomiting episodes: improved rate after session 3	24/43 (55.81 %)	14/38 (36.84 %)	*
Zhang 2005	Acupuncture + moxibustion (AMC) *N* = 50	Chinese herbal medicine *N* = 50	NVP, ketones, electrolytes, rate of food intake: cured rate	21/50 (42 %)	9/50 (15.25 %)	
Zhang 2005	Acupuncture + moxibustion (AMC) *N* = 50	IV fluid therapy + conventional therapy *N* = 50	NVP, ketones, electrolytes, rate of food intake: cured rate	21/50 (42 %)	5/50 (9.09 %)	*
Liu 2007	Acupuncture + IV therapy *N* = 47	IV fluid therapy *N* = 47	Treatment effect: Nausea, vomiting, food intake	38/47 (80.85 %)	23/47 (48.93 %)	*
Wang 2008	Acupuncture + IV therapy *N* = 53	IV fluid therapy *N* = 42	Nausea, vomiting, electrolytes	41/53 (77.35 %)	17/42 (0.47 %)	*
Mao 2009	Acupuncture + IV therapy *N* = 30	IV fluid therapy + Chinese herbal medicine *N* = 30	Total treatment effect: electrolytes and vomiting rate: cured rate	27/30 (90 %)	3/30 (10 %)	
Mao 2009	Acupuncture + IV therapy *N* = 30	IV fluid therapy + conventional therapy *N* = 30	Ketones: cured rate	27/30 (90 %)	4/30 (13.33 %)	*
Liu 2011	TCM Acupuncture *N* = 30	TCM Moxibustion *N* = 30	Nausea and vomiting, ketones, rate of food intake: cured rate	20/30 (66.67 %)	19/30 (63.33 %)	*
Ma 2013	Acupuncture + IV therapy *N* = 30	IV fluid therapy *N* = 30	Total treatment effect: ketones, vomiting rate: cured rate	28/30 (93.3 %)	10/30 (33.33 %)	*
Ou 2001	Ear acupressure *N* = 30	Chinese herbal medicine *N* = 30	Total treatment effect: electrolytes, nausea and vomiting rate: cured rate	3/30 (10.0 %)	3/30 (10.0 %)	*
Fan 1995	TCM moxa *N* = 151	Chinese herbal decoction *N* = 151	Nausea and vomiting Total: Cured rate	146/151 (96.7 %)	89/151 (58.9 %)	*
Xu 2009	TCM moxa *N* = 26	IV fluid therapy *N* = 25	Nausea and vomiting, ketones, rate of food intake: Cured rate	17/26 (65.38 %)	9/25 (36.0 %)	*
Lu 2012	TCM Moxa + IV fluid + conventional therapy *N* = 32	IV fluid therapy + conventional therapy *N* = 32	Nausea and vomiting, ketones, rate of food intake: Cured rate	10/32 (31.25 %)	5/32 (15.62 %)	*

**Table 5:** Continuous outcomes on last day of treatment from original studies (*included in pooling)

Studies (author, year)	Number of subjects, intervention		Outcome measurement	Outcomes (mean + SD)		Included in pooling
	Treatment group	Comparator		Treatment group	Comparator	
Belluomini 1994	Acupressure P6 *N* = 30	Sham acupressure point *N* = 30	Rhodes Index scores	5.80 ± 2.9	7.04 ± 2.6	*
			Nausea scores:			
Belluomini 1994	Acupressure P6 *N* = 30	Sham acupressure point *N* = 30	Rhodes Index scores	1.28 ± 1.9	1.63 ± 2.3	*
			Emesis scores:			
Belluomini 1994	Acupressure P6 *N* = 30	Sham acupressure point *N* = 30	Total	8.69 ± 5.0	10.03 ± 4.6	*
Werntoft 2001	Acupressure P6 *N* = 20	Sham acupressure *N* = 20	VAS	4.2 ± 2.6	5.9 ± 2.4	*
			Degree of nausea			
Werntoft 2001	Acupressure P6 *N* = 20	No treatment *N* = 20	VAS	4.2 ± 2.6	6.5 ± 2.2	
			Mean degree of nausea			
Jamigorn 2007	Acupressure P6 + placebo tablets *N* = 33	Sham acupressure + Vit B6 *N* = 33	Rhodes index score, Improvement in nausea, vomiting and retching	4.1 ± 1.8	5.3 ± 2.1	*
Saberi 2013	Acupressure P6 *N* = 48	No treatment *N* = 45	Rhodes Index scores	4.25 ± 3.38	5.66 ± 3.10	*
			Vomiting			
			Nausea	8.03 ± 4.11	7.08 ± 3.0	*
			Retching	3.66 ± 2.47	4.48 ± 2.25	
			Total	14.56 ± 8.66	17.23 ± 6.91	*
Smith 2002	Traditional acupuncture *N* = 148	Sham Acupuncture *N* = 148	Nausea	3.4 ± 3.0	3.7 ± 2.8	*
			Dry retching	0.8 ± 1.4	0.9 ± 1.4	
			Vomiting	0.9 ± 1.5	1.0 ± 1.6	*
Smith 2002	TCM Acupuncture *N* = 148	Acupressure P6 *N* = 148	Rhodes Index scores:	3.4 ± 3.0	4.0 ± 3.3	
			Nausea			
			Dry retching	0.8 ± 1.4	0.9 ± 1.3	
			Vomting	0.9 ± 1.5	0.9 ± 1.8	
Smith 2002	Traditional acupuncture *N* = 148	No treatment *N* = 149	Rhodes Index scores	3.4 ± 3.0	5.0 ± 3.0	
			Nausea			
			Dry Retching	0.8 ± 1.4	1.6 ± 1.7	
			Vomiting	0.9 ± 1.5	1.4 ± 2.0	
Mao 2009	Acupuncture + IV therapy *N* = 30	IV fluid therapy + Chinese herbal medicine *N* = 30	Ketones	1.20 ± 0.41	1.53 ± 0.68	
Mao 2009	Acupuncture + IV therapy *N* = 30	IV fluid therapy + conventional therapy *N* = 30	Ketones	1.20 ± 0.41	1.60 ± 0.72	
Ou 2001	Ear acupressure *N* = 30	Chinese herbal medicine *N* = 30	Vomiting	3.53 ± 1.72	1.33 ± 1.69	*
			Ketones	1.47 ± 1.66	0.67 ± 1.32	
			Main symptoms	18.4 ± 11.02	12.13 ± 9.67	*
Puangsricharern 2008	Auricular acupressure *N* = 45	No treatment *N* = 46	Mean Rhodes index Nausea and vomiting scores	7.7 ± 4.9	11.3 ± 9.2	*

Data from nine studies could not be entered into the meta-analyses because the way the outcomes were presented did not allow pooling. Six of these studies [2, 20, 22, 31, 32, 39] reported a significant difference in the treatment group compared to their control group. Table 6 shows more detailed information of excluded studies, reasons for exclusion and significance according to the author. Eventually, 20 studies met the inclusion criteria for pooling.

**Table 6:** Studies excluded from analyzes because of insufficient information

Reason for exclusion	Study	Significance for treatment group according to author
No measure of variability	Shin 2007	- significant for degree of nausea and vomiting
		- significant reduction for ketonuria levels over time by women with HG.
	Li 2010	- significant difference (*P* <0.05) for the severity and frequency of nausea and vomiting).
		- ketone bodies disappeared in the 2 acupressure groups significantly faster (*p* < 0.05) than in the group with IV fluid therapy.
	Liu 2012	- a statistically significant difference (*P* <0.05) for the severity and frequency of nausea and vomiting compared to IV fluid therapy.
Data reported in Mean and interquartile range (IQR)	Rad 2012	- statistically significant difference favouring Youmen acupressure over sham acupressure
	Heazell	- no difference between length of stay, amount of medication, or fluid required between the acupressure and placebo groups
		- acupressure reduced the number of patients who stayed more than four nights in the hospital.
	Knight	- no statistically significant difference between the control and intervention groups.
Data reported in Mean rank	Steele	- The treatment group had significantly less frequency and severity of nausea and vomiting of pregnancy than the placebo group
	Can Gurkan	- Acupressure would appear to be effective in symptom control, and alleviation and placebo effects in reducing the symptoms of nausea and vomiting during pregnancy.
Data reported only means of error bar plots	O’Brien 1996	- No benefit of acupressure for symptom relief compared with either sham acupressure or no treatment

#### Analyses for dichotomous data

**Fig. 2:**
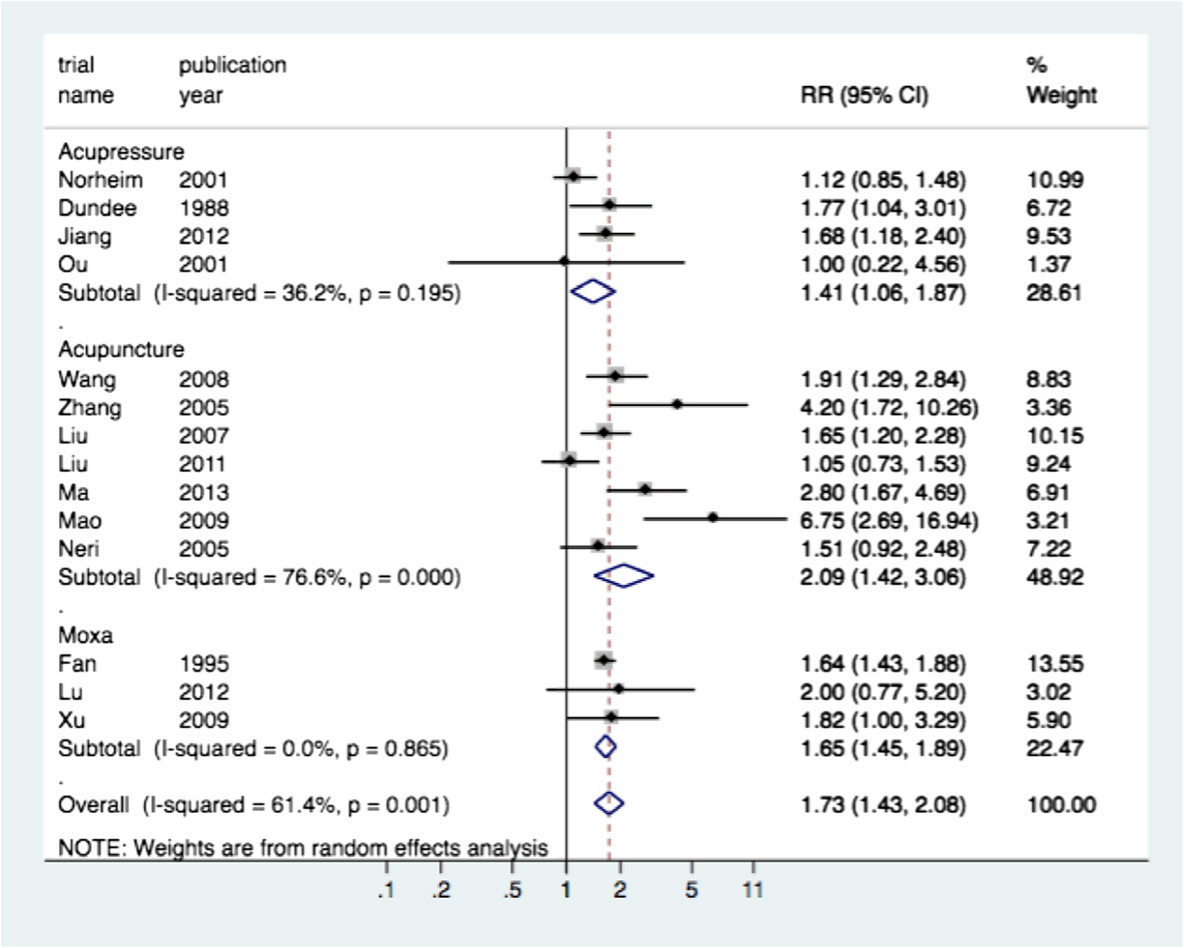
Improvement in nausea and vomiting during pregnancy per technique of acustimulation (relative risk (RR), 95 % CI)

Overall analysis for dichotomous data from 14 studies using acupressure [3, 36, 38, 42], acupuncture [8, 28–30, 33, 34, 41] or moxa [11–13] showed a beneficial reduction in the combined outcome for nausea, vomiting, and ketones in case of HG with a pooled RR of 1.73 (95 % CI 1.43 to 2.08, I squared 61 %) (Fig. 2), indicating 73 % fewer patients with symptoms at outcomes measured in the treatment group compared to those of the control group. Subgroup analyses of the various acustimulation techniques each show significant improvements with similar pooled RRs as combined analysis.

Acupressure [3, 37, 39, 42] reduced the severity of symptoms in NVP and HG by 41 % (RRp 1.41, 95 % CI 1.06 to 1.87, I squared 36 %). The effectiveness of acupuncture [11, 29–31, 34, 35, 41] was twice as large as the effect of the control group (RRp 2.09, 95 % CI 1.42 to 3.06, I squared 77 %) and moxibustion [11–13] improved symptoms by 65 % (RRp 1.65, 95 % CI 1.45 to 1.89, I squared 0 %).

#### Analyses for continuous data

**Fig. 3:**
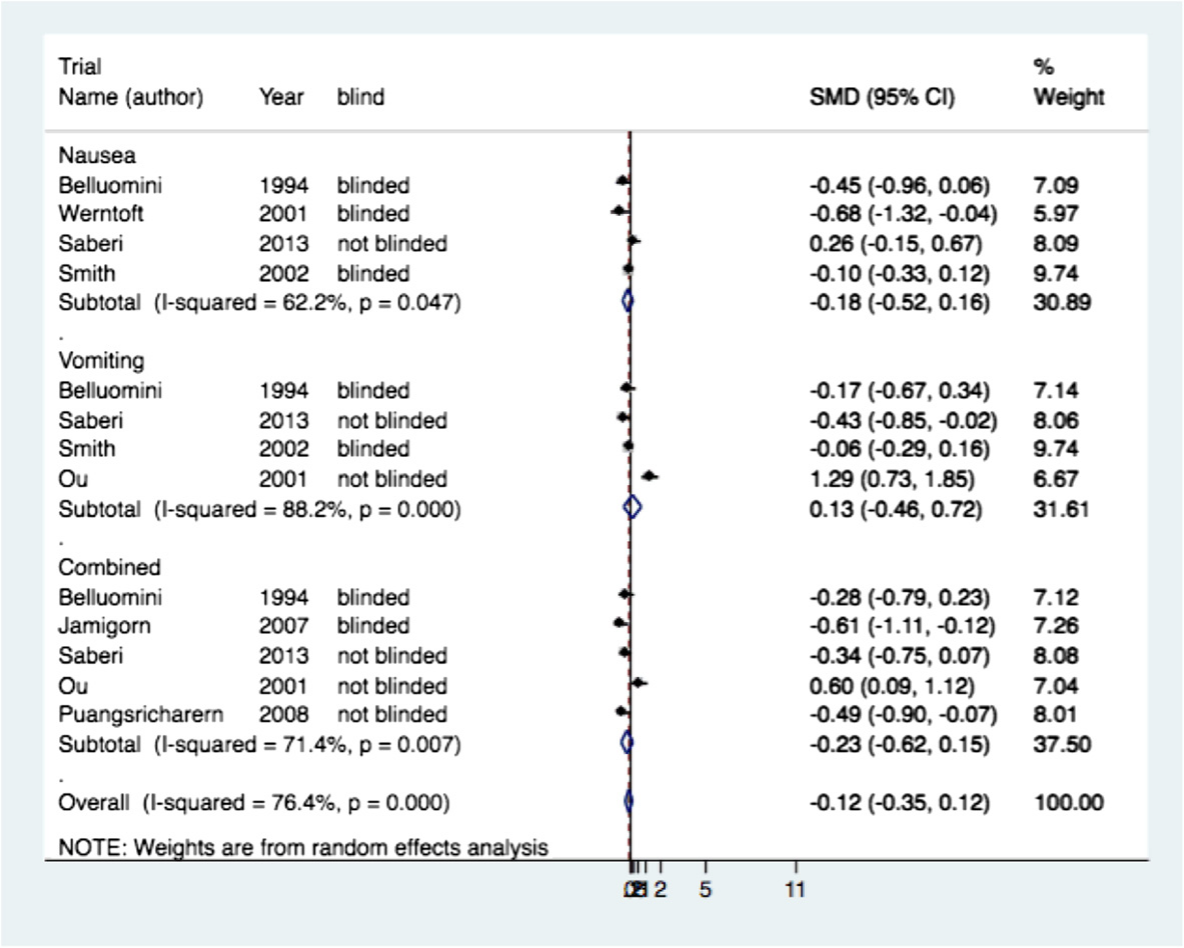
Efficacy per outcome measurement for studies with continuous outcome measures (Standard mean difference (SMD), 95 % CI)

Figure 3 shows the analyses for continuous data from seven studies [5–7, 10, 37, 40, 42]. Results are presented per outcome measurement for nausea, vomiting or combined. We did not include the results for retching, ketones and acupuncture against PC6 acupuncture.

Mean analysis from four studies [5, 6, 10, 37] did not show any evidence of an effect on nausea (pooled SMD −0.18, 95 % CI −0.52 to 0.16, I squared 62 %), nor did a similar analysis for vomiting from four studies [6, 10, 37] (pooled SMD 0.13, 95 % CI −0.46 to 0.72, I squared 88 %).

The analysis for the effect on the combined outcome for nausea and vomiting from five studies [6, 7, 37, 40, 42] did not show a significant effect of the treatment either (pooled SMD −0.23, 95 % CI −0.62 to 0.15, I squared 71.4 %). Overall SMD for all outcomes resulting from studies with continuous outcome measures was -0.12 (95 % CI −0.35 to 0.12, I-squared 76 %). There was no difference between blinded and non-blinded studies.

#### Sensitivity analysis

Four additional sensitivity analyses were perfomed, one excluding control groups with Chinese herbal medicine or conventional treatment [8, 11, 28, 33] and one restricting pooling to Chinese studies only. The results showed no differences with overall results. The third sensitivity analysis showed that, although there was some limited evidence for the effect of the stimulation of one point (RR = 1.43 (95 % CI 1.03 to 2.00, I squared 57 %), the use of a combination of acupoints according to TCM diagnoses yielded a better result in the treatment outcomes for NVP and HG (RR = 1.73 (95 % CI 1.43 to 2.08, I squared 62 %).

We performed a fourth sensitivity analysis to determine if the heterogeneity between studies could be explained by the different control groups. The crude RR for the studies with dichotomous data was 1.73 (95 % CI, 1.43 to 2.08). The RR, stratified by the therapy used in the control group did not alter the RR 1.78 (95 % CI 1.51 to 2.08). The crude pooled SMD for studies with continuous data was −0.23 (95 % CI −0.62 to 0.15) while the pooled SMD, stratified by the therapy used in the control group became statistically significant (pooled SMD −0.49, 95 % CI −0.65 to −0.34).

#### Adverse events reporting

Four studies [5, 35, 38, 40] reported on adverse events: increased sickness and local pain of the wrist due to tightness of the wrist band, and sleep disturbance, altered taste, bruising, pressure in the nose, headache and one case of increased sickness for acupuncture. No adverse effects were reported in studies for auricular acupuncture or moxibustion.

## Discussion

The present study reviewed randomized controlled trials on the efficacy of different techniques of acupoint stimulation for the treatment of NPVor HG in early pregnancy. To our knowledge, this is the first systematic review that also systematically included studies in Chinese for NVP or HG. The different acustimulation techniques examined here were acupressure finger or wrist band, auricular acupressure, traditional acupuncture and moxibustion. Most studies in the trials with acupressure were in English and examined the result of the stimulation of one point (PC6 or Neiguan) in studies for NVP. The studies for acupuncture and moxibustion were mainly conducted in Chinese for the treatment of HG using a combination of the following five points: Zu San Li (ST36), Nei Guan (PC6), Zhong Wan (CV12), Gong Sun (SP4) and San Yin Jiao (SP6).

Our meta-analysis included data from 20 trials. Mean analysis for nausea, vomiting and the combined effect from studies with continuous outcome measures did not show any evidence of symptom relief in NVP and HG. If control groups with Chinese herbal medicine in a sensitivity analysis were excluded, the effect on the combined outcome of nausea and vomiting compared to sham- or placebo-controlled intervention groups became significant (SMD −0.43, 95 % CI −0.65 to −0.2, I squared 0 %). Although there was a statistical difference between the two groups, the decrease was not clinically relevant. The overall analysis for dichotomous data showed a beneficial reduction in the combined outcome for nausea, vomiting, and ketones in case of HG (RR = 1.73 (95 % CI 1.43 to 2.08, I squared 61 %). We could not perform subgroup analyses by blinding because of poor or unclear blinding in most Chinese studies.

We are aware that we did not include all data, given the fact that we excluded control groups that were not optimally blinded. Exclusion of data from studies with multiple arms results might be a potential bias. We have made this decision because data from studies that are not blinded are less reliable. Moreover, it is not appropriate to include multiple comparisons in a meta-analysis, because every patient would be counted multiple times. Nevertheless, results obtained in this meta-analysis should be interpreted with caution. A major limitation in this study was that we faced a considerable amount of statistical heterogeneity among the trials. This might be due to the combination of data from trials on different interventions, different comparison groups, and a lack of standardization of primary outcomes measured or reported. In addition, for the sensitivity analyses we combined trials with the same control group and this did not reduce statistical heterogeneity. Moreover, the methodological quality of the included studies assessed by the Cochrane’s risk of bias tool was mixed. Some studies had high rates of attrition, poor allocation concealment and other methodological problems, which put them at high risk of bias. Another major limitation was blinding, especially in the Chinese studies. Although many of the included studies were described as being double-blind or as having kept women blind to group allocation, lack of effective blinding may also have introduced bias. Some of the trials that investigated the effectiveness of blinding provided some evidence that women may have had some idea of group allocation [10, 35, 38]. Lack of blinding or unconvincing blinding may be particularly relevant where the main outcome is women’s subjective, self-reported symptoms.

According to the revised STRICTA criteria, some essential details of the acupuncture treatment protocol were often insufficiently described. This is not an unexpected finding, given the fact that STRICTA was introduced in 2010. Precise description of these components of the acupuncture procedures will enable other researchers to replicate and evaluate the reported treatment protocol critically, accurately and reliably in both research and clinical settings [19].

Very few studies in the current review reported adverse events for the treatment with acupressure and acupuncture. No serious adverse events were reported and none for auricular acupressure and moxibustion. Although it may not be safe to assume that because negative outcomes were not reported, they did not occur, a systematic review about adverse events following acupuncture [43] suggested that most adverse events can easily be avoided by standardizing teaching and clinical practices.

A previous meta-analysis [4] and two recent reviews [1, 17] on acustimulation effects for NVP showed limited evidence for the effects of PC6 acupressure or acupuncture for reducing NVP. No trials of treatments for HG showed any evidence of benefit. In contrast with the latest Cochrane review [1], which tried to present findings for a time point approximately three days after the start of treatment, we opted to choose the last day of the intervention for outcome measurement. Hence, we sometimes obtained a different result for some studies in both reviews.

## Conclusion

Although there is some evidence that different acustimulation techniques significantly reduce the combined outcome for nausea, vomiting, and ketones in case of HG, it is too early to definitely conclude on the beneficial effects of acustimulation for the treatment of NVP and HG, taking into account the non-significant results in studies with continuous outcome measures and the moderate quality of the studies, especially with regard to blinding. Future clinical trials with a rigorous design and large sample sizes should be conducted to evaluate efficacy and safety of these interventions for NVP and HG.
